# Aurora Kinase B Inhibition: A Potential Therapeutic Strategy for Cancer

**DOI:** 10.3390/molecules26071981

**Published:** 2021-04-01

**Authors:** Naheed Arfin Borah, Mamatha M. Reddy

**Affiliations:** 1 The Operation Eyesight Universal Institute for Eye Cancer, L.V. Prasad Eye Institute, Bhubaneswar 751024, India; naheed.borah@gmail.com; 2 School of Biotechnology, KIIT University, Bhubaneswar 751024, India

**Keywords:** aurora kinase B (AURKB), cancer, AURKB regulation, AURKB inhibitors, therapy related drug resistance, combination therapy

## Abstract

Aurora kinase B (AURKB) is a mitotic serine/threonine protein kinase that belongs to the aurora kinase family along with aurora kinase A (AURKA) and aurora kinase C (AURKC). AURKB is a member of the chromosomal passenger protein complex and plays a role in cell cycle progression. Deregulation of AURKB is observed in several tumors and its overexpression is frequently linked to tumor cell invasion, metastasis and drug resistance. AURKB has emerged as an attractive drug target leading to the development of small molecule inhibitors. This review summarizes recent findings pertaining to the role of AURKB in tumor development, therapy related drug resistance, and its inhibition as a potential therapeutic strategy for cancer. We discuss AURKB inhibitors that are in preclinical and clinical development and combination studies of AURKB inhibition with other therapeutic strategies.

## 1. Introduction

Aurora kinases (AURKs) are protein serine/threonine kinases consisting of three members in the gene family—aurora kinase A (AURKA), aurora kinase B (AURKB) and aurora kinase C (AURKC) [1]. AURKs are critical regulators of the cell cycle, with AURKA and AURKB playing a key function in mitosis [1], whereas AURKC has a significant role in gametogenesis [2,3]. AURKs have three different domains, of which the kinase domain shares a high degree of homology among all the members [4]. The functions of AURKs are well-defined by their localization and spatio-temporal expression [5] and also the sequence differences in their N-terminal region [4]. Overexpression of AURKs in tumors has been shown to trigger aneuploidy and genomic instability [6] that leads to tumor development, invasion and metastasis. AURKA and its functions have been widely investigated and several reviews were authored to highlight its importance in cancer [7,8]. The role of AURKC in cancer is not completely understood and needs further investigations. This review focuses on AURKB, a potential drug target from the AURK gene family.

Aurora kinase B is encoded by the AURKB gene located on chromosome 17 and is also known by other names including AIK2, AIM1, ARK2, AIRK2, IPL1, STK1, STK5 and STK12. AURKB along with other AURKs plays a vital role in the regulation of cell cycle. Phosphorylation of histone H3 by aurora kinases is essential for chromosome segregation during cell division. Both AURKA and AURKB have been shown to phosphorylate histone H3 [9]. The role of elevated AURKB in increasing the phosphorylation of histone H3 on Ser10 and aneuploidy has been elucidated in studies involving Chinese hamster embryo cells exogenously overexpressing AURKB. The function of AURKB in transformation was further demonstrated in vivo by injecting these AURKB overexpressing cells into BALB/c nu/nu mice [10]. Induced AURKB expression is also linked to tumorigenesis mediated by H-Ras [11].

Based on its overexpression in various tumors, AURKB has emerged as an important drug target. Small molecule inhibitors were designed to specifically inhibit AURKB function in various tumors. In this review, we focus on AURKB and its function and deregulation in tumorigenesis. Further, we discuss AURKB inhibition as a promising therapeutic strategy and AURKB inhibitors that are in different phases of clinical development. Additionally, the use of AURKB inhibitors in combination with other therapeutic targets is discussed.

## 2. Structure and Function of AURKB

**Figure 1:**
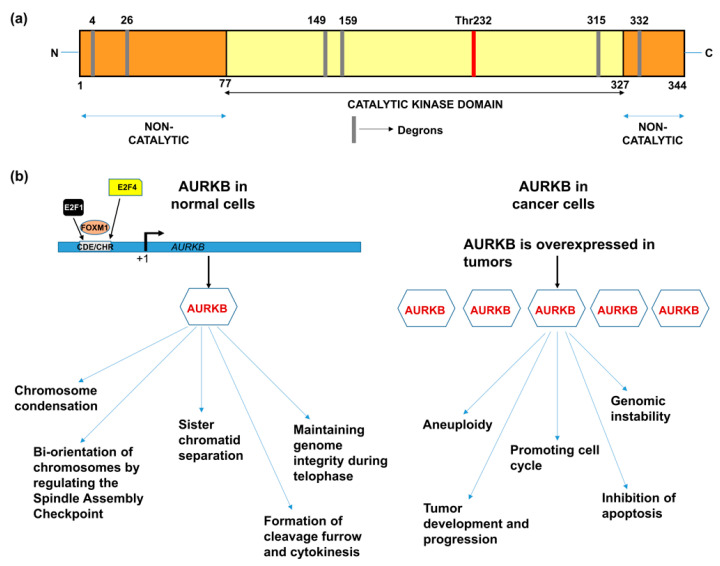
(**a**) Domain structure of AURKB showing catalytic and non-catalytic domains [14,16]. (**b**) Functions of AURKB in normal cells and cancer cells [8,14].

AURKB, similar to other members of the aurora kinase gene family, is composed of three domains: (i) N-terminal domain, (ii) kinase domain, and (iii) C–terminal domain [12]. The kinase domain or catalytic domain is highly conserved between all the three members of the aurora family, whereas the N-terminal domain shows a varied degree of sequence dissimilarity that provides selectivity for protein-protein interactions. The kinase domain in AURKB is composed of a β-stranded lobe on the N-terminal side and an α-helical lobe on the C-terminal side. These two lobes are connected together by a hinge region, which permits active kinase conformation [13]. The C-terminal lobe of the kinase domain contains the catalytic T-loop whose auto-phosphorylation at Thr232 results in AURKB activation. AURKB contains three types of degrons; KEN motif, D-box and DAD/A box that are thought to mediate its degradation. The catalytic domain contains D-boxes (reviewed in [14]). The non-catalytic N-terminal domain has a KEN motif and a DAD/A box, whereas the C-terminal domain has a D-box. However, Nguyen et al., has showed that only N-terminal KEN and DAD/A boxes are responsible for AURKB degradation [15] (Figure 1a).

The binding of E2F1, E2F4, FoxM1 and DP-2 to the AURKB promoter transcriptionally regulates AURKB. These transcription factors bind to the cell cycle-dependent element (CDE) and cell cycle gene homology region (CHR) located up-stream of the transcription start-site (Figure 1b; reviewed in [14]). AURKB is an essential member of the chromosomal passenger complex (CPC) which additionally includes INCENP, survivin and borealin [17,18]. AURKB is located on the chromatin before the onset of mitosis and promotes chromosome condensation by the phosphorylation of histone H3 and centrosome protein A (CENP-A) [19]. In pro-metaphase, AURKB, as part of the CPC, moves towards the kinetochores and is associated with repairing faulty spindle kinetochore attachments (spindle assembly checkpoint) [20]. AURKB ensures adequate alignment and segregation of sister chromatids and relocates to the microtubules during the transition from metaphase to anaphase [21]. AURKB regulates the distribution of Kif-2A and limits Kif-2A-controlled depolymerization of microtubules, thereby, mediating microtubule formation and functioning [22]. AURKB has also been implicated in cytokinesis, explained by its presence at the mid-body during telophase [23,24,25].

## 3. Deregulation of AURKB in Cancer

**Figure 2:**
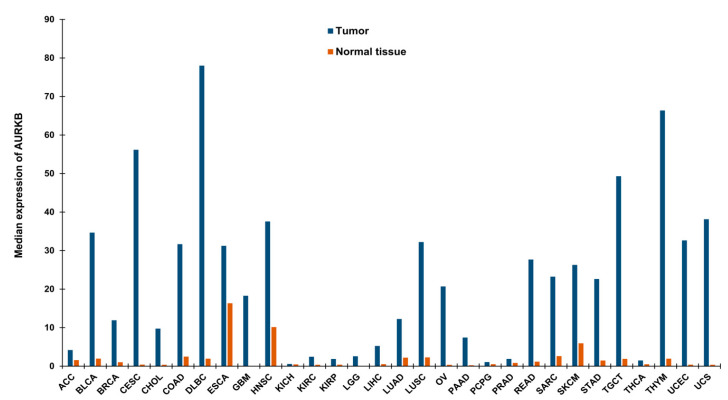
Median expression of AURKB in a variety of tumors and their respective normal tissues. The data has been retrieved from GEPIA [43]. ACC—Adrenocortical carcinoma, BLCA—Bladder Urothelial Carcinoma, BRCA—Breast invasive carcinoma, CESC—Cervical squamous cell carcinoma and endocervical adenocarcinoma, CHOL—Cholangio carcinoma, COAD—Colon adenocarcinoma, DLBC—Lymphoid Neoplasm Diffuse Large B-cell Lymphoma, ESCA—Esophageal carcinoma, GBM—Glioblastoma multiforme, HNSC—Head and Neck squamous cell carcinoma, KICH—Kidney Chromophobe, KIRC—Kidney renal clear cell carcinoma, KIRP—Kidney renal papillary cell carcinoma, LGG—Brain Lower Grade Glioma, LIHC—Liver hepatocellular carcinoma, LUAD—Lung adenocarcinoma, LUSC—Lung squamous cell carcinoma, OV—Ovarian serous cystadenocarcinoma, PAAD—Pancreatic adenocarcinoma, PCPG—Pheochromocytoma and Paraganglioma, PRAD—Prostate adenocarcinoma, READ—Rectum adenocarcinoma, SARC—Sarcoma, SKCM —Skin Cutaneous Melanoma, STAD—Stomach adenocarcinoma, TGCT—Testicular Germ Cell Tumors, THCA—Thyroid carcinoma, THYM—Thymoma, UCEC—Uterine Corpus Endometrial Carcinoma, UCS—Uterine Carcinosarcoma.

Drawing parallels with the functions of AURKB in mitosis, it is anticipated that alterations of AURKB either as amplification or overexpression could provide a proliferative advantage to cancer cells (Figure 1b). In fact, its role in tumor cell transformation was clarified by overexpressing AURKB in murine epithelial cells. When these AURKB overexpressing cells were injected into nude mice, formation of mammary epithelial tumors was observed along with amplifications and deletions of DNA isolated from these mouse mammary tumors [26]. Over the years, aberrant AURKB expression has been reported in a variety of malignancies including human seminoma [27], and thyroid carcinoma [28]. Using mRNA expression analysis Smith et al., found that AURKB expression is markedly elevated in non-small cell lung carcinoma (NSCLC) in comparison to matched untransformed lung tissues. This study also showed that AURKB overexpression led to poor progression free survival, however overall survival was not affected [29]. In contrast to this finding, a study by Vischioni et al., has found that AURKB expression is significantly associated with older age at diagnosis and reduced overall survival in adenocarcinoma subtype of NSCLC [30]. Likewise, AURKB was found to be induced in the squamous carcinoma subtype of NSCLC and the expression level of AURKB also served as a marker for resistance to paclitaxel, a drug commonly used in the treatment of NSCLC [31]. Similar to NSCLC, metastatic colorectal cancer patients lived significantly shorter when they had high levels of AURKB expression in their tumor tissues [32] and the publicly available databases further showed that increased AURKB expression also correlated significantly with reduced survival in breast cancer patients [33]. AURKB expression has been identified as a prognostic biomarker in glioblastoma [34], gastric cancer [35] and oral cancer [36,37,38]. AURKB overexpression was found to be increased in prostate cancer tissues compared to healthy controls [39]. Hepatocellular carcinoma tissues also showed significantly elevated mRNA expression of AURKB compared to paired healthy liver tissues and was found to be an independent prognostic marker for tumor invasiveness and prognosis [40]. Pediatric acute lymphoblastic leukemia and acute myeloid leukemia (AML) exhibited high levels of both AURKA and AURKB compared to control bone marrow mononuclear cells; however, the inhibition of AURKB alone resulted in apoptosis suggesting that AURKB is a putative drug target but not AURKA in these hematologic malignancies [41]. Recently, we have shown that AURKB is overexpressed in retinoblastoma compared to adjacent healthy retina and its expression significantly correlated with histological risk factors such as optic nerve and anterior chamber invasion [42]. Additionally, we have retrieved expression data of AURKB from Gene Expression Profiling Interactive Analysis (GEPIA) database [43] for a variety of cancers and compared it to their respective normal tissue counterparts (Figure 2). The above studies demonstrating the overexpression of AURKB in tumors compared to their non-cancerous counterpart tissues suggest the possible therapeutic targeting of AURKB in tumors.

## 4. Regulation of AURKB Function in Cancer

Over the years it has been shown that AURKB is overexpressed in various tumors and contributes to tumor development and progression. Several mechanisms underlying the regulation of AURKB and its interactions with other oncogenes or tumor suppressors are currently being explored. AURKB is regulated by upstream activators such as Myc, and cyclin K and it also regulates functions of certain proteins such as c-Myc and p53.

### 4.1. AURKB Regulation by Myc and Vice-Versa

The Myc oncogenes (c-MYC, MYCN, MYCL) are important determinants of tumor progression in malignancies driven by their overexpression or amplification. den Hollander et al., reported that c-MYC promotes expression of both AURKA and AURKB in c-Myc mediated B-cell lymphoma [44]; however, the regulation of AURKB was rather indirect [44]. In our recent study, we showed enrichment of a MYCN binding motif on the promoter of AURKB in human retinoblastoma and that supports a direct regulation of AURKB by MYCN [42]. Previously, MYCN was shown to be a direct transcriptional regulator of AURKB in neuroblastoma [45]. Interestingly, Jiang et al., delineated that AURKB stabilizes c-MYC in T-cell acute lymphoblastic leukemia (T-ALL) by phosphorylating at Ser67. c-MYC then activates AURKB transcription, creating a positive feedback loop, in turn switching-on a cascade of oncogenic interactions leading to T-cell leukemogenesis [46]. Oncogenes such as MYCN also regulate the expression of a few other dysregulated genes such as enzymes involved in altered metabolism in tumor cells [47,48].

### 4.2. Bcr-Abl Positively Regulates AURKB

The Bcr-Abl oncoprotein is a protein tyrosine kinase associated with chronic myeloid leukemia (CML) and ALL. Yang et al. have reported that Bcr-Abl induces the expression of both AURKA and AURKB through Akt signaling [49].

### 4.3. AURKB Crosstalks with BRCA1 and BRCA2

The inactivation of BRCA1 and/or BRCA2 induces tumor development. Wang et al. described that there is a cross talk between BRCA1/2 and AURKB wherein they inversely control tumor proliferation and tetraploidy of tumor cells. This implies that AURKB disruption leads to a decrease in cell proliferation and cytokinesis whereas disruption in BRCA1/BRCA2 resulted in abnormal cytokinesis, eventually, encouraging tumor progression. It is further considered that this interplay may be through the action of p53 and cyclin A [50].

### 4.4. RASSF7 Activates AURKB

Ras association domain-containing protein 7 (RASSF7) has been previously described as an important mitotic protein and shown to be upregulated in various cancers namely islet cell tumors, ovarian clear cell carcinoma, endometrial cancer and pancreatic ductal carcinoma (reviewed in [51]). Along with activation of AURKB, RASSF7 has a significant contribution in regulating the microtubule cytoskeleton [52]. Further, it was shown that RASSF7 downregulation leads to a loss of AURKB activation in cancer cells [52].

### 4.5. p53 Dependent Tumor Suppressor FBXW7 Negatively Regulates AURKB

F-box and WD repeat containing 7 (FBXW7) protein is a component of the E3 ubiquitin ligase complex and known p53-dependent tumor suppressor [53]. Previously it has been shown that FBXW7 is mutated in breast, bladder and cervical cancers (reviewed in [54]). Mutations or diminished levels of p53 result in increased expression of miR-25, which in turn leads to decreased levels of FBXW7 and the subsequent increase in AURKA levels [55]. Similarly, it was demonstrated that FBXW7 is a negative regulator of AURKB [56].

**Figure 3:**
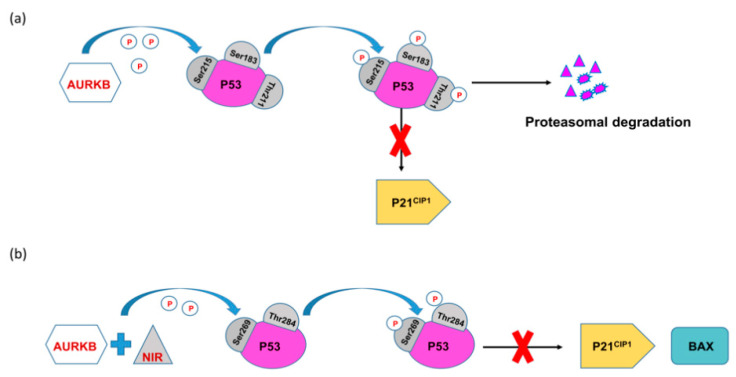
(**a**) Regulation of p53 by AURKB. (**b**) p53 regulation by AURKB and novel inhibitor of histone acetyltransferase repressor (NIR).

AURKB in turn suppresses the activity of p53 by phosphorylation at Ser183, Thr211, and Ser215 which quickens its degradation by the proteasome. As a result, the expression of p21Cip1, which is a known cell cycle inhibitor and downstream target of p53, goes down. Inhibition of AURKB was shown to restore the expression of p53 and its targets (Figure 3a) [57]. The above studies indicate a potential feedback loop between p53, FBXW7 and AURKB.

Additionally, it has been reported that AURKB in association with novel inhibitor of histone acetyltransferase repressor (NIR), phosphorylates p53 at Ser269 or Thr284 and greatly depletes its transcriptional activity, thereby, compromising its downstream targets p21 and Bax (Figure 3b) [58]. AURKB also contributes to Epstein-Barr Virus (EBV) induced B-cell oncogenic transformation by downregulating the activity of p53 homolog p73 while functioning along with the latently expressed viral membrane protein, Epstein-Barr nuclear antigen 3C (EBNA3C) [59].

### 4.6. AURKB Regulation by MDM2

MDM2 is an E3 ubiquitin ligase that is shown to be an important oncoprotein in various cancers. MDM2 exerts its functions both through and independent of p53. Recently, using PCR array experiments and MDM2 inhibitor Nutlin-3, it was observed that MDM2 modulates cell cycle possibly through AURKB-CDK1 signaling pathway [60].

### 4.7. AURKB Is a Downstream Target of Cyclin K in Prostate Cancer

Cyclin K is a member of the cyclin family of transcription regulators which function by association with cyclin dependent kinases. In prostate cancer, cyclin K was shown to mediate proliferation and inhibit apoptosis likely through AURKB [61].

## 5. Targeting AURKB in Cancer

Deregulated expression of AURKs is increasingly viewed as a potential drug target. AURKs are successfully inhibited in several preclinical cell line and animal models. A variety of small molecule inhibitors to target AURKs have been developed and are in different phases of clinical trials. The development of AURKA inhibitors and their progress has been described recently [7]. The possibility of targeting AURKB has gained momentum during last few years.

Taking into account that the members of the aurora kinase family have very high homology in the kinase domain, most small molecule inhibitors developed against aurora kinases have overlapping inhibitory activity. Despite this, pioneering collaborations between industry and academia led to the development of hesperadin and ZM447439, the former being predominantly selective for AURKB. ZM447439 was further modified to develop AZD1152 [62]. Most of the inhibitors were synthesized with an aim to improve therapy, but over the years, they have been extensively used to understand the varied functions and complex regulations mediated by AURKB. In the following section, we discuss some of the AURKB inhibitors that have high selectivity for AURKB and have undergone clinical trials.

### 5.1. AURKB Specific Inhibitors

#### 5.1.1. Hesperadin

Hesperadin is an indolinone-based ATP-competitive AURKB inhibitor with an IC_50_ of 250 nM in cell free assays [63]. The indolinone moiety of hesperadin binds to the catalytic cleft of the active enzyme. The forces of interaction between the two molecules are through hydrogen bonding and van-der Waals contact [64]. Further research on hesperadin analogues has revealed that additional hydrogen bonding by lipophilic substitution in the indolinone core could confer enhanced stability and activity to the drug [65]. It has been previously shown that hesperadin causes abnormal mitosis and impairment in cytokinesis. HeLa cells treated with hesperidin do not proliferate and become polyploid in nature [65].

#### 5.1.2. Barasertib

Barasertib is an ATP-competitive AURKB inhibitor developed by optimizing ZM447439. It is also known as AZD1152, AZD1152-HQPA and AZD2811. The novel acetanilide-substituted pyrazole-aminoquinazoline prodrug efficiently gets converted to the active form AZD1152-hydroxyquinazoline pyrazol anilide (AZD1152-HQPA) that lacks the phosphate group found in the pro drug. Barasertib is highly selective for AURKB with an IC_50_ value of 0.37 nM in cell free assays. It shows a 1000-fold more selectivity for AURKB when compared with AURKA [16]. Over the years, barasertib has been extensively tested in a variety of tumors and has emerged as a lead therapeutic molecule.

Barasertib has been shown to inhibit cell proliferation, induce polyploidy and subsequently increase apoptosis in AML cell lines. Also, the efficacy of chemotherapy agents was potentiated by AZD1152 in murine xenograft models [66]. Moreover, barasertib was used to target AURKB in NSCLC that had acquired resistance to anti-EGFR therapy [67]. In small cell lung cancer (SCLC) with high amplification of the MYC family proteins, AZD1152 inhibited tumor growth in-vivo [68]. Further, AURKB inhibition by AZD1152 hindered the growth of human lung, colon and hematologic malignancy xenografts in immunodeficient mice [69]. Apart from pre-clinical studies, AZD1152 has been tested in a number of clinical trials to study its efficacy and safety profile predominantly in AML. A study conducted in Japanese patients reported an overall hematologic response rate of 19%. This was a promising result considering no dose limiting toxicities were reported with neutropenia and febrile neutropenia being the most common adverse events [70]. Further, the pharmacokinetics, metabolism and excretion of barasertib was assessed and it was reported that the rate of clearance was slow, and the drug was excreted mostly through feces [71].

A study comparing the responses of barasertib and low dose of cytosine arabinoside (LDAC) as monotherapy was conducted on AML patients. LDAC has been shown to have therapeutic benefits in patients unable to go through intensive chemotherapy. The primary endpoint of the study was the objective complete response rate (OCRR), which is defined as the proportion of patients reaching complete response (CR). A CR is measured according to the criteria established by the international working group for AML trials [72]. Treatment with barasertib showed a significant improvement in OCRR (35.4%) in comparison to LDAC (11.5%). The median overall survival for barasertib was found to be 8.2 months vs 4.5 months for LDAC. This study has shown that barasertib treatment was beneficial, albeit with an increased but manageable toxicity [73]. A combination treatment of barasertib with LDAC was performed in 22 patients with AML. An overall response rate of 45% (n = 10/22) was achieved with two patients reporting dose limiting toxicities [74].

Additionally, barasertib was tested on patients with solid tumors and in relapsed/refractory diffuse B-cell lymphoma [NCT01354392]. In case of solid tumors, neutropenia was reported as the most common dose-limiting toxicity and the toxicity profile was found to be tolerable. The recorded responses were at best modest [75,76]. The report on B-cell lymphoma described that AZD1152 induced a short-lived reduction in tumor. However, the combination of inconvenience in administering the drug and modest responses did not warrant further investigation of AZD1152 in the treatment of B-cell lymphoma [77].

Overall, the clinical trials established the proof-of concept that AURKB could possibly be targeted with AZD1152 (Table 1). Majority of the reports suggested a positive response to the treatment and a manageable toxicity profile with neutropenia being the most common adverse event, but, it is also known that barasertib at higher concentrations inhibits FLT3 and KIT kinases required for hematopoiesis and hence may result in dose-limiting neutropenia [78,79]. However, the mode of administering the drug, which is intravenous infusion for a period of 4 days in diffuse B-cell lymphoma [77] and 7 days for AML [80], has made it highly inconvenient. This led to the development of AZD2811(formerly known as AZD1152) nanoparticle formulation which exceeded the anti-tumor activity reported for AZD1152 drug alone. The improved activity of the nanoparticle formulation was attributed to increased inhibition of phospho-histone H3, polyploidy and apoptosis [80,81]. The details of the clinical trials with AZD2811 nanoparticle formulation are included in Table 1. The IC50s of various cell lines [82,83,84,85,86,87,88,89,90,91] that have been tested with AZD1152 are included in Table S1.

#### 5.1.3. SP-96

This is a quinazoline-based AURKB inhibitor with an IC_50_ of 0.316 nM and is the first described non-ATP competitive inhibitor against AURKB. It has been shown to inhibit the triple negative breast cancer cell line MDA-MD-468 [78]. SP-96 is 2000-fold more selective for AURKB in comparison to FLT3 or KIT. It is known that barasertib at higher concentrations also inhibits FLT3 and KIT [78,79]. Both FLT3 and KIT kinases play an important role in hematopoiesis and their inhibition may result in neutropenia as observed in the clinical trials for barasertib. Hence, SP-96 plausibly can reduce the adverse effects caused by barasertib [78].

### 5.2. Pan Aurora Kinase Inhibitors in Clinical Trials

#### 5.2.1. GSK1070916

It is an azaindole-based ATP-competitive inhibitor that is highly selective for AURKB and AURKC with IC_50_ values of 0.38 and 1.5 nM, respectively. It is more than 250-fold more selective for AURKB when compared with AURKA. The discovery of GSK1070916 was initiated by optimizing a series of 7-azaindole based molecules in which cellular activity was enhanced by introducing a 2-aryl group onto the azaindole. Further, treatment of A549 human lung cancer cell lines with GSK1070916 produced a half-maximal effective concentration of 7 nM [113]. GSK1070916 has been shown to inhibit proliferation of tumor cells in more than 100 human tumor cell lines with IC50 values of <10 nM [114]. The IC50s of various cell lines tested in pre-clinical studies [115,116] with GSK10710916 have been shown in Table S1. It also shows anti-tumor activity in human tumor xenograft models including breast, colon and lung cancer [114]. A phase 1 clinical trial sponsored by Cancer Research UK has been conducted with GSK1070916 in patients suffering from advanced solid tumors. [NCT01118611]. The maximum tolerated dose was determined to be 85 mg/m^2^/day with neutropenia as the dose-limiting toxicity [94].

#### 5.2.2. Danusertib (PHA-739358)

Danusertib is a 3-aminopyrazole-derived pan-aurora kinase inhibitor with IC_50_s of 13, 79 and 61 nM in AURKA, AURKB and AURKC, respectively. It has been shown that danusertib induces apoptosis, cell cycle arrest and autophagy in ovarian cancer cells [117]. Additionally, danusertib can inhibit growth of liver metastases both in vitro and in vivo, in gastroenteropancreatic neuroendocrine tumors [118]. The IC50s of danusertib for various cell lines have been reported [119,120,121,122,123,124,125,126,127] and summarized in Table S1. A phase 1 clinical trial of danusertib in patients with advanced solid tumors showed satisfactory tolerance with preliminary indications of anti-tumor activity [102].

T315I mutation in Bcr/Abl confers resistance to treatment with Bcr/Abl inhibitors in ALL patients with Philadelphia chromosome (Ph). Fei et al., showed that treatment of Ph positive ALL cells carrying T315I mutation with danusertib can be an alternate therapeutic strategy, especially for imatinib-, nilotinib- or dasatinib-resistant tumors [128]. A phase 1 clinical trial with 37 patients (22 with advanced stage CML and 15 with Ph positive ALL) was conducted for danusertib. The results showed an acceptable toxicity profile with promising anti-tumor activity [103]. Phase 2 clinical studies have also been conducted for danusertib and details of all the clinical trials have been summarized in Table 1.

#### 5.2.3. AT9283

1-Cyclopropyl-3-(3-(5-(morpholinomethyl)-1*H*-benzo[d]imidazol-2-yl)-1*H*-pyrazol-4-yl)urea is a pan-aurora kinase inhibitor that shows similar selectivity for AURKA and AURKB with an IC50 of 3 nM. AT9283 is also effective against additional kinases such as Janus kinases (JAKs) and Abl (T315I) [129]. It was shown to inhibit AURKB activity, induce endoreduplication, suppress cell proliferation and enhance apoptosis in B-cell non-Hodgkin’s lymphoma cell lines. Additionally, AT9283 represses tumor growth in mice xenograft models [130]. Pre-clinical studies with AT9283 have been reviewed in Mills, et al., [131]. A recent report showed that AT9283 exhibits anti-proliferative activity in tyrosine kinase inhibitor resistant CML [132].

A phase 1 trial conducted in advanced malignancies showed that AT9283 was well tolerated, and the recommended dose for phase 2 trial was determined to be 40 mg/m^2^/day administered at day 1 and day 8 every 21 days. The dose limiting toxicities were febrile neutropenia and neutropenia [95]. A phase 1 trial in leukemia patients reported myocardial infarction, cardiomyopathy, hypertension, pneumonia and multiple organ failure as dose limiting toxicities, thus, suggesting extensive cardiovascular monitoring in further studies [97]. A phase 2 study in multiple myeloma reported that no objective responses were observed in the treated patients. They suggested that AT9283 was not recommended for further study for treating multiple myeloma, but the limitations of the trial could not warrant firm conclusions [96]. Overall, AT9283 showed a manageable toxicity profile in clinical trials but further studies are essential to determine its efficacy in clinical use. The list of clinical trials conducted with AT9283 have been summarized in Table 1. The IC50s of AT9283 in CML cell lines are shown in Table S1. [133]

#### 5.2.4. AMG900

AMG900 is *N*-(4-(3-(2-Aminopyrimidin-4-yl)pyridin-2-yloxy)phenyl)-4-(4-methylthiophen-2-yl)phthalazin-1-amine, a highly selective pan-AURK inhibitor with IC_50_s of 5, 4 and 1 nM for AURKA, AURKB and AURKC respectively. It is a phthalazinamine-based compound which competitively inhibits binding of ATP to the active site of aurora kinases [134]. Reports have shown that AMG900 inhibited the growth of glioblastoma cells in vitro ultimately leading to cell cycle arrest and senescence [135]. In MOLM-13 AML cell line, treatment with AMG900 was linked to inhibition of histone H3 phosphorylation, polyploidy and increased apoptosis with the upregulation of p53 [136]. The IC50 concentrations of AMG900 on breast cancer cell lines are shown in Table S1 [137]. 

In a phase 1 clinical trial with AML patients, the safety and efficacy of AMG900 was investigated. Nausea, diarrhea, febrile neutropenia and fatigue were the most common adverse events with 9% of patients showing complete response. Additionally, the study reported that patients with higher baseline expression of AURKA, TTK, CDC2, BIRC5 and CCNB1 were more susceptible to showing a positive outcome [106]. In a different phase 1 study, AMG900 was shown to be rapidly absorbed with quick clearance. The maximum tolerated dose was determined with or without granulocyte-colony stimulating factor (G-CSF) and found to be 40 and 25 mg/day, respectively. Overall, AMG900 showed a manageable toxicity when used with G-CSF with neutropenia being the most common adverse event [107].

#### 5.2.5. CYC116

CYC116 (4-methyl-5-(2-(4-morpholinophenylamino)pyrimidin-4-yl)thiazol-2-amine) is an orally bioavailable panAURK inhibitor which is derived from *N*-phenyl-4-(thiazol-5-yl) pyrimidin-2-amine with IC_50_ values of 8 and 9.2 nM for AURKA and AURKB, respectively. CYC116 has been shown to suppress histone H3 phosphorylation and induce polyploidy [138]. A clinical trial for CYC116 was subsequently started in patients with advanced solid tumors but was terminated due to a decision taken by the sponsors Cyclacel Pharmaceuticals, Inc. (Berkeley Heights, NJ, USA) The trial was aimed at examining the safety profile of CYC116 (NCT00560716).

#### 5.2.6. Other Pan-AURK Inhibitors

**Figure 4:**
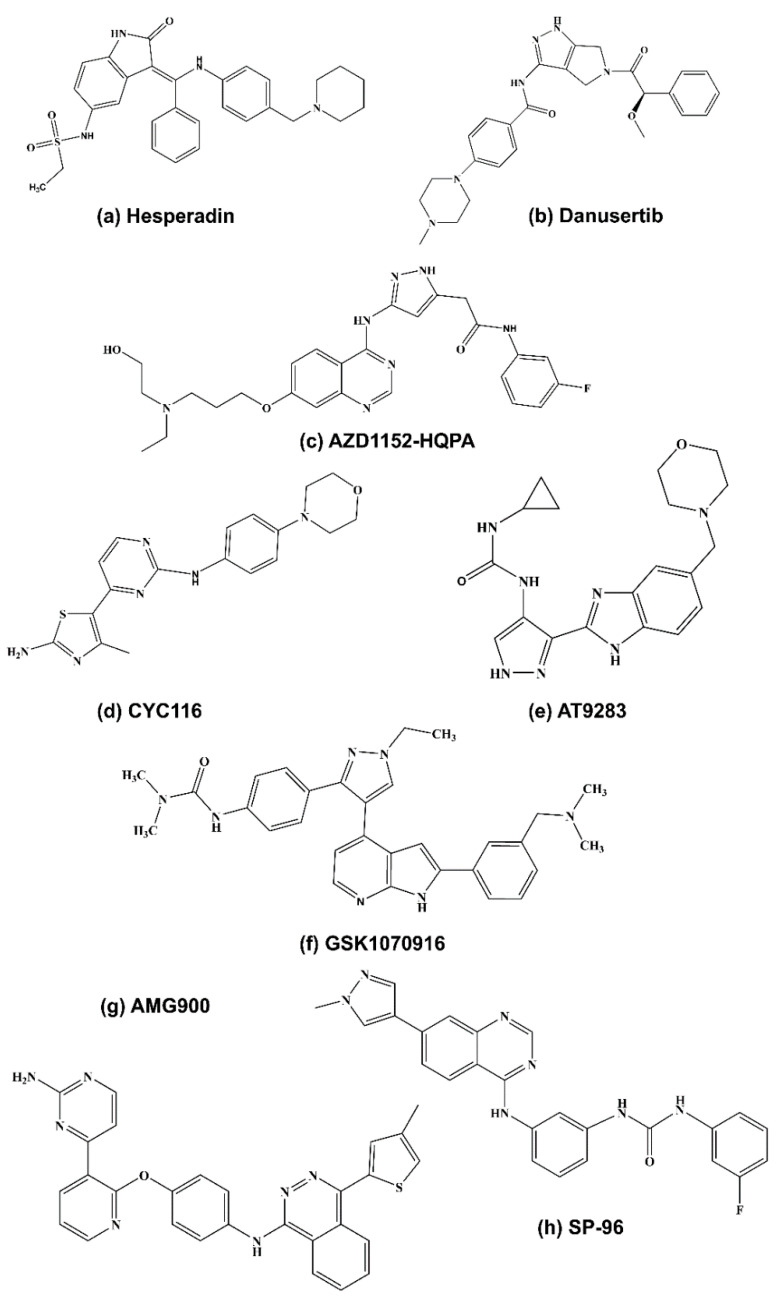
Structure of AURKB inhibitors: (**a**) hesperadin [16], (**b**) danusertib [151] (**c**) AZD1152-HQPA [152], (**d**) CYC116 [151], (**e**) AT9283 [153], (**f**) GSK1070916 [16], (**g**) AMG900 [151], (**h**) SP-96 [78].

Additionally, PHA680632 [139], reversine [33,140], CCT129202 [141], CCT137690 [142,143], SNS-314 [144], quercetin [145,146] are pan-aurora kinase inhibitors that have been tested in pre-clinical studies. Furthermore, VX-680 [147,148,149] and BI 811283 [108,109] are pan-AURK inhibitors that have been studied in clinical trials. VX-680 has been extensively reviewed in Pinel, et al., and Portella, et al., [16,150]. The details of clinical trials with BI 811283 are included in the Table 1. The IC_50_s pertaining to pre-clinical studies with AURKB inhibitors are summarized in Table S1. The structures of the described inhibitors are shown in Figure 4.

## 6. Therapy-Related Drug Resistance and AURKB

Therapy-related drug resistance and the resultant tumor progression are major causes of poor prognosis in various cancers. The resistant tumors usually develop mutations in certain oncogenes or tumor suppressors, or certain genes are expressed at abnormally high levels. AURKB expression has been linked to therapy related drug resistance in different malignancies including vemurafenib-resistant melanoma [154], temozolomide-resistant glioblastoma [154], and epidermal growth factor receptor tyrosine kinase inhibitor-resistant NSCLC [67]. Head and neck squamous cell carcinoma cells exhibiting resistance to cetuximab were found to show elevated expression of AURKB on a microarray analysis [155]. Breast cancer cells resistant to the drug fulvestrant showed increased AURKB phosphorylation and AURKB inhibitor barasertib preferentially reduced growth of tumor cells resistant to fulvestrant and tamoxifen [156]. Similarly, MCF7 breast cancer cells resistant to aromatase inhibitors when screened with 195 compounds identified AURKs as novel drug targets. These aromatase resistant breast cancer cells also showed significant growth inhibition when treated with barasertib (specific to AURKB) and JNJ-7706621 (AURKA/B and CDK inhibitor) [157]. Likewise, CML cells resistant to tyrosine kinase inhibition specifically showed reduction in cell proliferation with dual ABL and AURKB inhibitors PHA-739358 and R763/AS703569 [158]. Also, HI-511, a dual inhibitor of AURKB and BRAF V600E achieved drug sensitivity in both susceptible and resistant melanoma cell growth [154]. Overexpression of AURKB in response to various chemotherapeutic drugs suggests that targeting AURKB would be a strategy to overcome therapy-related drug resistance.

On the contrary, mutations in AURKB kinase domain were also identified. T-cell ALL cells were modeled to study the in vitro drug resistance mechanism against AURKB inhibition. When the cells were exposed to high concentrations of ZM447439, a Gly160Glu mutation was observed in the kinase domain of AURKB that could prevent the inhibitor binding in addition to other AURKB independent mechanisms at further high concentrations [159]. Using barasertib-resistant pancreatic carcinoma cell lines and microarray analysis, Guo et al., have shown that elevated expression of multi-drug resistant protein (MDR1) and breast cancer resistant protein (BCRP) is responsible for drug resistance and their expression could serve as a marker for barasertib sensitivity [160].

In some of the studies, it has been shown that AURKB inhibition should not be combined with certain drugs. Treatment with barasertib along with paclitaxel enhanced resistance to paclitaxel treatment in NSCLC cell lines in a dose-dependent manner. In addition, these studies showed that taxanes should not be used if patients express high levels of AURKB [31]. Therefore, expression of AURKB can be used as a predictive biomarker for treatment of NSCLC patients with taxanes.

Using a mutation-prone cell line, Girdler et al., have shown that point mutations can also arise in ATP-binding pocket of AURKB against treatment with AURKB inhibitor ZM447439 [161]. This study suggests that mutations in response to AURKB inhibition might develop similar to other kinase inhibitors and thus additional studies are warranted to understand drug resistance mechanism and subsequent development of novel inhibitors or combination strategies to overcome the therapy-mediated resistance.

## 7. Combination Therapy with AURKB Inhibition

Aurora kinase B inhibition has achieved good in vitro and in vivo efficacy in pre-clinical models. Currently, some AURKB inhibitors are in clinical trials, but have not yet reached the clinic. The combination studies involving AURKB inhibitors with other anticancer drugs hold promise and efforts were made in that direction using in vitro and in vivo models. The efficacy of AURKB inhibition along with other therapeutic strategies was initially tested using cell line models and the pan-AURK inhibitor VX-680 in combination with chemotherapy drug doxorubicin. VX-680 reduced cell viability of C1A, PC3 and LNCaP cells. The decrease in cell viability was elevated when VX-680 was used in combination with doxorubicin. In addition, VX-680 was shown to sensitize PC3 cells for treatment with doxorubicin [162]. Another pan-AURK inhibitor CCT137690 demonstrated synergistic anti-oral cancer activity when used in combination with EGFR inhibitor gefitinib or PI-3K inhibitor pictilisib [143]. Subsequently, several studies attempted combination therapies along with AURKB inhibition. Barasertib augmented the therapeutic response of vincristine and daunorubicin in AML cell lines as well as mouse models [66] and a topoisomerase I inhibitor CPT-11 in HCT-116 colorectal carcinoma cells [163]. Combination targeting of AURKB and orally available BH3 mimetic, ABT-263 led to a decrease in cell viability in several different tumor cell lines compared to monotherapy with VX-680. siRNA knockdown with AURKA and AURKB further confirmed that synergistic activity was due to inhibition of AURKB [164]. Similar to studies above, AURKB inhibitor barasertib was shown to enhance combination effectiveness of oxaliplatin and gemcitabine in colon and pancreatic cancer respectively [155]. In vitro and in vivo analysis demonstrated that AURKB inhibition along with inhibition of DNA repair protein PARP1 had synergistic activity against skin cutaneous melanoma cells [165]. However, AURKB inhibitor barasertib and cytarabine used in combination exerted a greater-than-additive cytotoxicity in AML cells [166].

Recently, a few other studies attempted to identify the synthetic lethal interactions with AURKB inhibition. One of the studies using CRISPR/Cas9 identified that haspin kinase when inhibited in combination with AURKB inhibition considerably enhanced the antitumor activity in a synthetic lethal manner [147]. A different study using CRISPR/Cas9 parallelly identified that SCLC cells with loss of RB1 tumor suppressor gene are hyper-dependent on AURKB for their survival and inhibition with AURKB inhibitors significantly reduced cell proliferation demonstrating that loss of RB1 is synthetic lethal with AURKB inhibition [167]. AURKB inhibition was also found to be synthetic lethal with Myc overexpression [168,169]. Similarly, TAK-901, AURKB inhibitor from Takeda Pharmaceuticals showed synthetic lethal activity along with BCL-XL inhibition [170]. Further, loss-of-function RNAi screen identified that AURKB inhibition along with rapamycin had a synergistic activity in breast cancer cell lines [171]. Additional approaches including pharmacological small molecule inhibitor screens were employed to identify combination therapy treatments. One such screen identified synergistic efficacy of AURKB inhibitor Barasertib when combined with focal adhesion kinase inhibitors PF-562271 and VS-4718 to inhibit Ewing’s sarcoma cell growth [172].

AT9283 suppresses tumor growth in aggressive B-cell lymphomas and in these cells, it had a potent anti-AURKB activity. When AT9283 was used in combination with a taxane docetaxel, it showed a synergistic anti-tumor activity [130]. On the contrary, AT9283 showed both anti-AURKA and AURKB activity in multiple myeloma cells. The antimyeloma activity of AT9283 was further enhanced in a synergistic manner when used in combination with lenalidomide, a dicarboximide used in the treatment of multiple myeloma [153].

AURKB inhibition in combination with other drugs was successfully tested as a strategy to mitigate therapy-related drug resistance. Inhibition of AURKB using siRNA along with temozolomide enhanced the in vitro chemotherapeutic response of temozolomide-resistant glioma cells [173]. Nanoparticles of AURKB siRNA also showed comparable synergistic activity when used in combination with temozolomide in glioblastoma and enhanced survival of orthotopic mouse models of glioblastoma [174]. In a different study, Alafate et al., have performed kinome analysis and identified that AURKB is an important candidate responsible for the chemoresistance in temozolomide-resistant glioblastoma cells and the associated drug resistance could be mitigated by combined targeting of AURKB along with temozolomide [175]. Similarly, inhibition of AURKs enhanced the chemosensitivity to temozolomide and caused radio-sensitization in glioblastoma cells [176]. To incorporate AURKB inhibitors into clinical practice, additional studies involving exhaustive combination therapies should be tested, particularly, for the chemotherapy resistant tumors with elevated levels of AURKB expression.

## 8. Computational Chemistry Approaches to Develop Promising Inhibitors for AURKB

In the last few decades computational chemistry approaches have helped accelerate the process of drug development for various cancers. Molecular docking studies were employed to predict more efficient inhibitors against oncogenic protein kinases including aurora kinases. A recent in-silico analysis using docking-based comparative intermolecular contacts analysis (dbCICA) identified a lead molecule 85 (NCI 14040). The molecule 85 was further validated for its in vitro efficacy against AURKA. The study showed that the compound 85 has activity against pancreas, breast and prostate cancer cell lines [177]. 8-amino-substituted purine-based derivatives were synthesized to inhibit AURKs and the in vitro efficacy analysis against various tumor cell lines further identified that breast cancer cell lines were more sensitive compared to other cell lines tested [178]. In a different study, 4-anilinoquinoline derivatives with sulfonamide moiety were synthesized to inhibit AURKA/B. Docking studies were employed to confirm the molecular interactions between the inhibitors and the AURKs. The in vitro analyses identified that compound 9d among different synthesized molecules was more effective [179]. Further, Fernandes et al., using the Molegro Virtual Docker (MVD) software identified that IAF79 compound is a promising dual AURKB and FLT3 inhibitor for the treatment against various cancer types particularly for AML [180]. All the above studies highlight the role of computational approaches in drug development, and this specifically speeds up the process for rational drug design.

## 9. Conclusions

Deregulation of cell cycle plays a critical role in tumor initiation, progression, invasion and metastasis and the proteins involved in the regulation of cell cycle including AURKB are overexpressed in various tumors. AURKB promotes tumorigenesis and chemotherapy associated drug resistance. The expression of AURKB is regulated by other proteins such c-Myc, MDM2, MYCN, and cyclin K. Targeting AURKB is increasingly seen as a feasible therapeutic strategy against various tumors. Nevertheless, the currently available AURKB inhibitors though showed in vitro and in vivo therapeutic efficacy, they have not reached the clinic so far. Combination therapy of AURKB inhibition along with other small molecule inhibitors with activity against tumors or traditional chemotherapy agents is the need of the hour and should be pursued rapidly to achieve additional armamentarium in the fight against cancers. Further, several of the AURKB inhibitors have off-target effects on other kinases; and therefore, we need to develop and test more specific AURKB inhibitors for future use in fight against cancer.

## Figures and Tables

**Table 1:** List of clinical trials with AURKB inhibitors. Part of the data for the clinical trials have been extracted from clinicaltrials.gov (accessed on 29 March 2021).

Sl. No.	Drug	Study	Tumor	Phase	Sponsored by	Remarks	References/Clinical Trials.Gov Identifier
1	AZD1152	A Phase I, Open Label, Multi-centre Study to Assess the Safety, Tolerability, and Pharmacokinetics of AZD1152 in Japanese Patients With Acute Myeloid Leukaemia.	Leukemia	1	AstraZeneca (Cambridge, UK)	Promising response rate of 19% (3/16 patients) indicating the requirement of additional studies.	[70]/ NCT00530699
2	AZD1152	A Phase 2 Trial of AZD1152 in Relapsed/Refractory Diffuse Large B-cell Lymphoma	Lymphoma	2	Oxford University Hospitals NHS Trust (Oxford, England)	Although, AURKB appears to be a valid target, the relatively low responses and difficulty in administering makes AZD1152 an unsuitable candidate for monotherapy.	[77]/ NCT01354392
3	AZD1152	A Phase I, Open-Label, Multi-Centre Study to Assess the Safety, Tolerability and Pharmacokinetics of AZD1152 Given as a 2-h or 48-h Intravenous Infusions in Patients With Advanced Solid Malignancies	Solid tumors	1	AstraZeneca	Manageable tolerance with neutropenia and leukopenia.	[76]/ NCT00338182
4	AZD1152	A Phase I Open, Non-randomised, Single-centre Study to Assess the Metabolism, Excretion and Pharmacokinetics of AZD1152 and AZD1152 hQPA Following Intravenous Administration of [14C]-AZD1152 in Patients With Acute Myeloid Leukaemia (AML)	Leukemia	1	AstraZeneca	The drug was well tolerated in the tested population and excreted via hepatic metabolic routes. Potential benefits can be achieved with further investigations.	[71]/ NCT01019161
5	AZD1152	A Phase I/II, Open Label, Multi-centre Study to Assess the Safety, Tolerability, Pharmacokinetics and Efficacy of AZD1152 in Patients With Acute Myeloid Leukaemia.	Leukemia	1	AstraZeneca	A manageable toxicity profile was observed with a response rate of 25%	[92]/ NCT00497991
6	AZD1152	A Phase I, Open-label, Multi-centre, Multiple Ascending Dose Study to Assess the Safety and Tolerability of AZD1152 in Combination With Low Dose Cytosine Arabinoside (LDAC) in Patients With Acute Myeloid Leukaemia (AML)	Leukemia	1	AstraZeneca	The combination of Barasertib with low dose cytosine arabinoside showed acceptable tolerability with an overall response rate of 45% at the maximum tolerated dose	[74,93]/ NCT00926731
7	AZD1152	A Randomised, Open-label, Multi-centre, 2-stage, Parallel Group Study to Assess the Efficacy, Safety and Tolerability of AZD1152 Alone and in Combination With Low Dose Cytosine Arabinoside (LDAC) in Comparison With LDAC Alone in Patients Aged ≥ 60 with Newly Diagnosed Acute Myeloid Leukaemia (AML)	Leukemia	2/3	AstraZeneca	AZD1152 shows a significant improvement in response when compared to low-dose cytosine arabinoside with relatively high but manageable safety profile.	[73,93]/ NCT00952588
8	AZD1152	A Phase I, Open-Label, Multi-Centre Study to Assess the Safety, Tolerability and Pharmacokinetics of AZD1152 Given as a Continuous 7-Day Intravenous Infusion in Patients With Advanced Solid Malignancies	Solid tumors	1	AstraZeneca	The study was discontinued because of technical difficulties in administering the drug and lack of efficacy. Additionally, the prescribed schedule was inconvenient.	[76]/ NCT00497679
9	AZD1152	A Phase I, Open-Label, Multi-Centre Study to Assess the Safety, Tolerability and Pharmacokinetics of AZD1152 Given as a 2 Hour Intravenous Infusion on Two Dose Schedules in Patients With Advanced Solid Malignancies	Solid tumors	1	AstraZeneca	The study was terminated because of lack of efficacy of AZD1152 in monotherapy on solid tumors at the time of study	[75]/ NCT00497731
10	AZD1152	A Phase I/II, Open-Label, Multicentre 2-Part Study to Assess the Safety, Tolerability, Pharmacokinetics, and Efficacy of AZD2811 as Monotherapy or in Combination in Treatment-Naïve or Relapsed/Refractory Acute Myeloid Leukaemia Patients Not Eligible for Intensive Induction Therapy.	Leukemia	1/2	AstraZeneca	The study is currently in the recruitment phase.	NCT03217838
11	GSK1070916	A Cancer Research UK Phase I Trial to Evaluate Safety, Tolerability, Pharmacokinetics and Pharmacodynamics of Aurora B Inhibitor GSK1070916A in Patients With Advanced Solid Tumors.	Solid tumors	1	Cancer Research UK (London, UK)	Neutropenia was the dose limiting toxicity with 85 mg/m^2^/day being the maximum tolerated dose.	[94]/ NCT01118611
12	AT9283	A Phase I Study of AT9283 Given As a 24-h Infusion on Days 1 and 8 Every Three Weeks in Patients with Advanced Incurable Malignancy	Non-Hodgkin’s lymphoma and solid tumors	1	NCIC Clinical Trials group (Kingston, Canada)	AT9283 showed manageable tolerability with recommended phase 2 dose at 40 mg/m^2^/day given on day 1 and 8 every 21 days. The dose limiting toxicity was febrile neutropenia.	[95]/ NCT00443976
13	AT9283	A Phase II Study of AT9283 in Patients with Relapsed or Refractory Multiple Myeloma	Multiple myeloma	2	NCIC Clinical Trials group	The study reports that the dose and schedule of AT9283 used in the study is not recommended for further investigation for the treatment of multiple myeloma. Although, aurora kinases as a possible drug target is not ruled out.	[96]/ NCT01145989
14	AT9283	A Phase I/IIa Open-label Study to Assess the Safety, Tolerability and Preliminary Efficacy of AT9283, a Small Molecule Inhibitor of Aurora Kinases, in Patients With Refractory Hematological Malignancies	Leukemia	1/2	Astex Pharmaceuticals, Inc. (Pleasanton, CA, USA)	The study reports cardiac tachyarrythmias and severe reversible cardiomyopathy in addition to other toxicities associated with cytotoxic therapy. Reduction of leukemic blasts were observed in some patients but this did not lead to a significant clinical response.	[97]/ NCT00522990
15	AT9283	A Cancer Research UK Phase I/IIa Trial of AT9283 (A Selective Inhibitor of Aurora Kinases) Given Over 72 h Every 21 Days Via Intravenous Infusion in Children and Adolescents Aged 6 Months to 18 Years With Relapsed and Refractory Acute Leukemia	Leukemia	1	Cancer Research UK	The study shows that although toxicity was tolerable, there was no evidence suggesting efficacy of AT9283.	[98]/ NCT01431664
16	AT9283	A phase I dose escalation study of AT9283, a small molecule inhibitor of aurora kinases, in patients with advanced solid malignancies	Solid tumors	1	Astex Therapeutics Ltd. (Pleasanton, CA, USA); Cancer Research UK; Experimental Cancer Medicine Centre (UK); National Institute for Health Research Biomedical Research Centre (UK)	AT9283 was well tolerated up to a maximum tolerated dose of 27 mg/m^2^/72 h and febrile neutropenia was the dose limiting toxicity.	[99]
17	AT9283	A Phase I Trial of AT9283 (a Selective Inhibitor of Aurora Kinases) in Children and Adolescents with Solid Tumors: A Cancer Research UK Study	Solid tumors	1	Experimental Cancer Medicine Network (UK); Cancer Research UK; the Oak Foundation at The Royal Marsden Hospital (London, UK); National Institute for Health Research Biomedical Research Centres; Children’s Cancer and Leukemia Group (Leicester, UK)	AT9283 had manageable toxicity and was well tolerated.	[100,101]/ NCT00985868
18	PHA-739358	An Exploratory Phase II Study of PHA-739358 in Patients With Multiple Myeloma Harbouring the t(4;14) Translocation With or Without FGFR3 Expression	Multiple myeloma	2	Nerviano Medical Sciences (Milan, Italy)	The study was terminated due to low recruitment rate.	NCT00872300
19	PHA-739358	A Pilot Phase II Study of PHA-739358 in Patients With Chronic Myeloid Leukemia Relapsing on Gleevec or c-ABL Therapy	Leukemia	2	Jonsson Comprehensive Cancer Center (Los Angeles, CA, USA)	Results have not been reported so far	NCT00335868
20	PHA-739358	A Phase I Dose-Escalation Study of danusertib (PHA-739358) Administered as a 24-h Infusion With and Without G-CSF in a 14-day Cycle in Patients with Advanced Solid Tumors	Solid tumors	1	National Cancer Institute (Bethesda, MD, USA)	The study concluded that it was safe to administer danusertib and the recommended phase 2 dose was determined.	[102]
21	PHA-739358	A phase I study of danusertib (PHA-739358) in adult patients with accelerated or blastic phase chronic myeloid leukemia and Philadelphia chromosome-positive acute lymphoblastic leukemia resistant or intolerant to imatinib and/or other second generation c-ABL therapy	Leukemia	1	Nerviano Medical Sciences	Danusertib treatment had an acceptable toxicity profile and could be a promising agent for malignancies associated with Bcr-Abl.	[103]
22	PHA-739358	Randomized phase II study of danusertib in patients with metastatic castration-resistant prostate cancer after docetaxel failure	Prostate cancer	2	Nerviano Medical Sciences	Drug was well-tolerated with neutropenia being the most common adverse event. Monotherapy with danusertib showed minimal efficacy and further studies are recommended.	[104]/NCT00766324
23	PHA-739358	Phase I Pharmacokinetic and Pharmacodynamic Study of the Aurora Kinase Inhibitor danusertib in Patients With Advanced or Metastatic Solid Tumors	Solid tumors	1	Nerviano Medical Sciences	The recommended phase 2 dose was determined in the study and neutropenia was reported as the dose limiting toxicity. However, it was short lasting and there were no reported non-hematologic toxicities.	[105]
24	AMG900	A Phase 1 Study Evaluating the Safety, Tolerability, Pharmacokinetics and Pharmacodynamics of Orally Administered AMG900 in Adult Subjects With Acute Myeloid Leukemia	Leukemia	1	Amgen (Thousand Oaks, CA, USA)	The study reported manageable hematologic toxicities but the patient response was modest. Dose escalation was hampered due to prolonged cytopenias.	[106]/ NCT01380756
25	AMG900	A Phase 1, First-in-Human Study Evaluating the Safety, Tolerability, Pharmacokinetics and Pharmacodynamics of Orally Administered AMG900 in Adult Subjects With Advanced Solid Tumors	Solid tumors	1	Amgen	AMG900 showed acceptable tolerance.	[107]/ NCT00858377
26	CYC116	A Phase I Pharmacologic Study of CYC116, an Oral Aurora Kinase Inhibitor, in Patients With Advanced Solid Tumors	Solid tumors	1	Cyclacel Pharmaceuticals, Inc.(Berkeley Heights, NJ, USA)	The study was terminated by the sponsors	NCT00560716
27	BI 811283	An Open Phase I Single Dose Escalation Study of Two Dosing Schedules of BI 811283 Administered Intravenously Over 24 h Continuous Infusion in Patients With Advanced Solid Tumours With Repeated Administration in Patients With Clinical Benefit	Solid tumors	1	Boehringer Ingelheim (Ingelheim am Rhein, Germany)	The study demonstrated a manageable toxicity profile with disease stabilization recorded for 19 patients. Although, the limited anti-cancer activity did not warrant further development of the drug as a monotherapy agent.	[108] NCT00701324
28	BI 811283	An Open Phase I/IIa Trial to Investigate the Maximum Tolerated Dose, Safety, Efficacy and Pharmacokinetics of BI 811283 in Combination With Cytarabine in Patients With Previously Untreated Acute Myeloid Leukaemia Ineligible for Intensive Treatment	Acute Myeloid Leukemia	2	Boehringer Ingelheim	An acceptable safety profile was demonstrated but the use of BI 811283 with LDAC did not show increased treatment efficacy in comparison to LDAC treatment in isolation.	[109] NCT00632749
29	AZD2811	A Phase I, Open-Label, Multicentre Dose Escalation Study to Assess the Safety, Tolerability, and Pharmacokinetics of AZD2811 in Patients With Advanced Solid Tumours.	Solid tumors	1	AstraZeneca	The study determined the maximum tolerable dose and the drug is in further investigation	[110] NCT02579226
30	AZD2811	Phase II, Single-arm Study of AZD2811 and Durvalumab (MEDI4736) Combination Therapy in Relapsed Small Cell Lung Cancer Subjects With c-MYC Expression [SUKSES-E]	Small cell lung cancer	2	Keunchil Park, Samsung Medical Center (Seoul, South Korea)	The study is in the recruitment phase	NCT04525391
31	AZD2811	Phase II, Single-arm Study of AZD 2811 Monotherapy in Relapsed Small Cell Lung Cancer Patients [SUKSES-N3]	Small cell lung cancer	2	Samsung Medical Center	The study was terminated as the purpose of the study was fulfilled earlier.	[111] NCT03366675
32	AZD2811	A Phase II Multicenter, Open-Label, Single Arm Study to Determine the Efficacy, Safety and Tolerability of AZD2811 and Durvalumab Combination as Maintenance Therapy After Induction With Platinum-Based Chemotherapy Combined With Durvalumab, for the First-Line Treatment of Patients With Extensive Stage Small-Cell Lung Cancer	Small cell lung cancer	2	AstraZeneca	New study. The recruitment has not started yet	NCT04745689
33	AZD2811	A Phase I/II, Open-Label, Multicentre 2-Part Study to Assess the Safety, Tolerability, Pharmacokinetics, and Efficacy of AZD2811 as Monotherapy or in Combination in Treatment-Naïve or Relapsed/Refractory Acute Myeloid Leukaemia Patients Not Eligible for Intensive Induction Therapy.	Acute Myeloid Leukemia	1/2	AstraZeneca	This is an ongoing study and the latest update suggests good tolerability of the drug. Dose escalations are currently being planned.	[112] NCT03217838

## Data Availability

The data for Figure 2 has been extracted from gepia.cancer-pku.cn accessed on 28 March 2021.

## Supplementary Materials

The following are available online. Table S1. IC50 values for AURKB inhibitors in preclinical studies.
