# Supplementary material for: Aurora Kinase B Inhibition: A Potential Therapeutic Strategy for Cancer
Source: Molecules. 2021 Apr 1;26(7):1981. doi: 10.3390/molecules26071981 (PMC8037052; doi:10.3390/molecules26071981)
Supplement: Supplementary file 1 [file molecules-26-01981-s001.pdf]

molecules

Table S1. IC50 values for AURKB inhibitors in preclinical studies.

Sl. No.	Drug	Cell line	Tumor	$IC_{50}/EC_{50}/GI_{50}$ (nM)	References (Listed at the end of main text)
1	AZD1152	LNCaP	Prostate	25	[82]
2		PC-3		10	
3	AZD1152	SW620	Colon	10.27	[69]
4	AZD1152	HGC27	Gastric	3.98	[83]
5		MGC803		2.82	
6	AZD1152	MOLM-13	Leukemia	12	[66]
7		MV-4-11		8	
8	AZD1152	JHH-1	Hepatocellular carcinoma	17.4	[84]
9		JHH-2		218	
10		JHH-4		155.6	
11		HuH-1		27.3	
12		HuH-6		3.7	
13		HuH-7		6.8	
14		HLE		45.9	
15		HLF		126.1	
16		PLC/PRF/5		76.9	
17		SK-Hep1		21.9	
18		Hep3B		7.6	
19		HepG2		14.7	
20	AZD1152	SW872	Liposarcoma	43.4	[85]
21		93T449		74.5	
22	AZD1152	HCT116	Colon cancer	20.12	[86]
23		HT-29		150	
24	AZD1152	A549	Lung	20.16	
25	AZD1152	MDA-MB-468	Breast	14	[87]
26		MDA-MB-435		125	
27		MDA-MB-231		105	
28		MDA-MB-361		70	
29		BT-474		8	
30	AZD1152	CAL-62	Thyroid	9.2	[88]
31		BHT-101		461.3	
32		8305C		287.8	
33		8505C		26.7	
34	AZD1152	SK-ES-1	Ewing sarcoma	5	[89]
35		A4573		6	
36		A673		5000	
37	AZD1152	TT	Thyroid	401.6	[90]
38	AZD1152	U2OS	Osteosarcoma	146	[91]
39	AZD1152	A549	Lung	0.9	[31]
40		SK-MES-1		1.2	
41		SK-LU-1		2.3	

42	AZD1152	HuH7	Hepatocellular carcinoma	16.72	[40]
43		Hep3B		4.79	
44		Y79	Retinoblastoma	39.38	[42]
45	GSK1070916	RPMI-8402	T-cell Acute Lymphoblastic leukemia	40	[115]
46		JURKAT		11260	
47		MOLT-4		4370	
48		CCRF-CEM		300	
49		PF-382		300	
50		HPB-ALL		20	
51		BE-13		20	
52	GSK1070916	Y79	Retinoblastoma	17.89	[42]
53	GSK1070916	KB-3-1	Cervical	16	[116]
54	GSK1070916	SW48	Colon	2	[114]
55		Colo201		2	
56		SW480		3	
57		WiDr		3	
58		Colo205		7	
59		RKO E6		7	
60		RKO		7	
61		LoVo		7	
62		HCT116		8	
63		SW620		8	
64		HT29		10	
65		SW1417		19	
66		DLD-1		28	
67		HCT-8		38	
68		Colo 320HSR		92	
69		Colo 320DM		96	
70		NCl-H630		127	
71	GSK1070916	U2OS	Osteosarcoma	34	[114]
72	GSK1070916	SK-BR-3	Breast cancer	4	[114]
73		MDA-MB-453		6	
74		MX-1		8	
75		MDA-MB-231		11	
76		MDA-MB-468		12	
77		MCF-7		13	
78		T47D		13	
79	GSK1070916	HELA	Cervical	13	[114]
80	GSK1070916	HN5	Head and Neck	10	[114]
81	GSK1070916	A498	Kidney	33	[114]
82		786-O		57	
83	GSK1070916	Hep3B	Hepatocellular carcinoma	12	[114]
84	GSK1070916	OVCAR-3	Ovarian	3	[114]
85		A2780		5	
86		OVCAR-4		11	
87		SKOV3		12	
88		OVCAR-8		30	

89	GSK1070916	AsPC-1	Pancreatic	2	[114]
90		Mia PaCa		3	
91		BxPC3		3	
92		PANC-1		13	
93	GSK1070916	PC3	Prostate	11	[114]
94		LNCaP		12	
95		DU145		15	
96	GSK1070916	SK-MEL-2	Skin	2	[114]
97		A375P		7	
98		SK-MEL-28		7	
99		SK-MEL-5		8	
100		SW954		11	
101	GSK1070916	HEC-1-B	Uterine	4	[114]
102	GSK1070916	NCI-H358	Lung	4	[114]
103		A549		7	
104		NCI-H157		8	
105		MV522		13	
106		NCI-H460		14	
107		NCI-H1299		23	
108		NCI-H1155		40	
109	GSK1070916	HUNS1	Myeloma	9	[114]
110		RPMI8266		11	
111		SKO 007		352	
112		U266B1		422	
113	GSK1070916	CRO-AP2	B-cell Lymphoma	3	[114]
114		CRO-AP5		3	
115		SR		3	
116		NU-DUL-1		4	
117		MHH-PREB-1		4	
118		OCI-LY-19		4	
119		SU-DHL-16		4	
120		Pfeiffer		4	
121		U-937		5	
122		SU-DHL-5		5	
123		SU-DHL-4		5	
124		SU-DHL-6		6	
125		HT		7	
126		JM1		7	
127		Farage		7	
128		MC/CAR		9	
129		RL		10	
130		DB		11	
131		SC-1		12	
132		DOHH-2		14	
133		Toledo		15	
134		SU-DHL-10		15	
135		ARH-77		15	

136		BC-1		18	
137		BC-3		27	
138		BCP-1		31	
139		RC-K8		38	
140		BC-2		75	
141		REC-1		77	
142	GSK1070916	ST486	Burkitt lymphoma	2	[114]
143		EB-2		2	
144		Raji		3	
145		GA10		4	
146		Daudi		5	
147		Jiyoye		5	
148		CA-46		5	
149		NAMALWA		6	
150		NC-37		7	
151		EB-1		7	
152		EB-3		7	
153		P3HR-1		8	
154		MC116		8	
155		1A2		9	
156		HS-Sultan		14	
157		DG-75		18	
158	GSK1070916	HD-MY-Z	Hodgkin lymphoma	4	[114]
159		Hs 445		5	
160		RPMI 6666		10	
161		L-428		31	
162		TO 175.t		54	
163	GSK1070916	JVM-3	B-cell Acute Lymphoblastic Leukemia	2	[114]
164		SUP-B15		2	
165		NALM-6		2	
166		KARPAS-231		3	
167		SEM		3	
168		RCH-ACV		4	
169		CESS		5	
170		Kasumi-2		6	
171		TANOUE		1478	
172	GSK1070916	PLB-985	Acute Myeloid Leukemia	1	[114]
173		NOMO-1		2	
174		CCRF-SB		2	
175		OCI-AML2		4	
176		OCI-AML3		4	
177		ML-2		5	
178		THP-1		5	
179		MV-4-11		7	
180		HL-60		10	
181		F-36P		10	
182		NB-4		11	

183		M-07e		13	
184		OCI-M1		20	
185		GDM-1		21	
186		BDCM		26	
187		CMK		27	
188		KG-1		32	
189		HEL 92.1.7		60	
190	GSK1070916	EM-2	Chronic Myeloid Leukemia	4	[114]
191		EM-3		4	
192		BV173		5	
193		KCL-22		5	
194		KU812		8	
195		K562		13	
196		MEG-01		44	
197	GSK1070916	ALL-SIL	T-cell Acute Lymphoblastic Leukemia	2	[114]
198		MOLT-16		3	
199		HSB-2		3	
200		CML-T1		3	
201		MPLT4		3	
202		JURKAT		3	
203		CTV-1		4	
204		SKW-3		5	
205		MOLT-3		5	
206		CEM/C1		6	
207		CCRF-CEM		7	
208		JRT3-T3.5		9	
209		DND-41		787	
210	GSK1070916	MJ	T-cell lymphoma	7	[114]
211		HuT 78		14	
212		HH		18	
213	GSK1070916	MEC-1	Chronic B-cell Leukemia	10	[114]
214	Danusertib (PHA-739358)	A375	Melanoma	254	[119]
215		SK-MEL-5		1718	
216	Danusertib (PHA-739358)	AGS	Gastric	1450	[120]
217		NCI-N78		2770	
218	Danusertib (PHA-739358)	Hep3B	Hepatocellular carcinoma	22030	[121]
219	Danusertib (PHA-739358)	KF62	Leukemia	200	[122]
220		HL60		3000	
221	Danusertib (PHA-739358)	C13	Ovarian	1830	[117]
222	Danusertib (PHA-739358)	MCF7	Breast	5140	[123]
223		MDA-MB-231		3090	
224	Danusertib (PHA-739358)	THP-1	Leukemia	2690	[124]
225		HL-60		3250	
226		K562		3020	
227		WM3211	Melanoma	1760	[125]

228	Danusertib (PHA-739358)	Lu1205		3340	
229		SK-MEL-28		12450	
230	Danusertib (PHA-739358)	T47D	Breast	210	[126]
231	Danusertib (PHA-739358)	Huh7	Hepatocellular carcinoma	15	[127]
232		HepG2		20	
233	AMG-900	HCC-1187	Breast cancer	0.08	[137]
234		MDA-MB-468		0.07	
235		HCC-38		0.04	
236		HCC-70		0.08	
237		EFM-19		0.39	
238		BT-20		0.46	
239		HCC-1395		0.5	
240		MDA-MB-157		0.45	
241		UACC-893		2.12	
242		MDA-MB-361		1.19	
243		CAMA-1		0.38	
244		BT-549		0.07	
245		ZR-75-1		0.07	
246		SUM-225		2.78	
247		UACC-732		0.7	
248		MDA-MB-415		2.23	
249		SK-BR-3		0.12	
250		BT-474		0.11	
251		HCC-1954		0.13	
252		MDA-MB-435		0.04	
253		HCC-1419		0.08	
254		MDA-MB-436		1.64	
255		Hs578T		0.92	
256		CAL-51		1.17	
257		MDA-MB-175		0.19	
258		SUM-190		0.27	
259		HCC-1806		0.02	
260		EFM-192A		0.09	
261		184A1		1.18	
262		184B5		1.03	
263		COLO-824		0.49	
264		DU-4475		1.15	
265		HCC-1143		0.03	
266		HCC-1937		0.06	
267		HCC-2218		2.05	
268		KPL-1		0.88	
269		MCF-10A		0.92	
270		MCF-7		0.22	
271		MDA-MB-231		0.25	
272		MDA-MB-453		0.02	
273		T47D		0.46	
274		UACC-812		15.21	

275	AMG-900	SW-872	Liposarcoma	3.7	[85]
276		93T449		6.5	
277	AT9283	BV173	Chronic Myeloid leukemia	55	[133]
278		KU812		26	
279		MYL		21	
280		KT-1		81	
281		KBM-5		84	
282		MEG-01		31	
